# Cancer-Associated Fibroblasts: Immunosuppressive Crosstalk with Tumor-Infiltrating Immune Cells and Implications for Therapeutic Resistance

**DOI:** 10.3390/cancers17152484

**Published:** 2025-07-28

**Authors:** Jogendra Singh Pawar, Md. Abdus Salam, Md. Shalman Uddin Dipto, Md. Yusuf Al-Amin, Moushumi Tabassoom Salam, Sagnik Sengupta, Smita Kumari, Lohitha Gujjari, Ganesh Yadagiri

**Affiliations:** 1 Division of Medicinal Chemistry and Pharmacognosy, College of Pharmacy, The Ohio State University, Columbus, OH 43210, USA; pawar.72@osu.edu (J.S.P.); kumari.45@osu.edu (S.K.); 2 The James Cancer Hospital and Solove Research Institute, Comprehensive Cancer Center, The Ohio State University, Columbus, OH 43210, USA; 3 Faculty of Biotechnology & Biomedical Engineering, Rajshahi Medical University, Rajshahi 6100, Bangladesh; 4 Department of Pharmacy, University of Rajshahi, Rajshahi 6205, Bangladesh; mddipto1001@gmail.com (M.S.U.D.); moushumi5350@gmail.com (M.T.S.); 5 Purdue University Interdisciplinary Life Sciences Graduate Program, Purdue University, West Lafayette, IN 47907, USA; amin50@purdue.edu; 6 Department of Chemistry, Purdue University, West Lafayette, IN 47907, USA; 7 Department of Radiology, University of Texas Southwestern Medical Center, Dallas, TX 75390, USA; sagnik.sengupta@utsouthwestern.edu; 8 Dorothy M. Davis Heart and Lung Research Institute, Division of Pulmonary, Critical Care & Sleep Medicine, Department of Internal Medicine, Wexner Medical Center, The Ohio State University, Columbus, OH 43210, USA; gujjari.lohitha@gmail.com; 9 Department of Obstetrics and Gynecology, College of Medicine, The Ohio State University, Columbus, OH 43210, USA

**Keywords:** cancer-associated fibroblasts, tumor-infiltrating immune cells, biological crosstalk, tumor microenvironment, CAF-induced immunosuppression, tumor cell resistance

## Abstract

Cancer is no longer considered as an isolated event. Rather, it occurs because of a complex biological drive orchestrating different cell types, growth factors, cytokines, and signaling pathways within the tumor microenvironment (TME). Cancer-associated fibroblasts (CAFs) are the most populous stromal cells within the complex ecosystem of TME, with significant heterogeneity and plasticity in origin and functional phenotypes. Very enigmatic cells, CAFs determine the progress and outcomes of tumors through extensive reciprocal signaling with different tumors infiltrating immune cells in the TME. In their biological drive, CAFs release numerous chemical mediators and utilize various signaling pathways to recruit and modulate tumor-infiltrating immune cells. The CAF-induced secretome and exosomes render immune cells ineffective for their antitumor activities. Moreover, by upregulating immune inhibitory checkpoints, CAFs create an immunosuppressive TME that impedes the susceptibility of tumor cells to tumor-infiltrating lymphocytes (TILs). Further, by depositing and remodeling extracellular matrix (ECM), CAFs reshape the TME, which enhances tumor growth, invasion, metastasis, and chemoresistance. Understanding of CAF biology and its crosstalk with tumor-infiltrating immune cells is crucial not only to gain insight in tumorigenesis but to optimize the potential of novel targeted immunotherapies for cancers. The complex relationships between CAFs and tumor-infiltrating immune cells remain unclear and need further study. Herein, in this narrative review we have focused on updates of CAF biology and its interactions with tumor-infiltrating immune cells in generating immunosuppressive TME and resistance to cell death.

## Simple Summary

Occurance of cancer and its outcomes are heavily influenced by its surroundings, known as the tumor microenvironment (TME). One key player in this environment is a type of cell called cancer-associated fibroblasts (CAFs). These cells are very active and influence how cancer behaves by interacting with the immune system. CAFs modulate tumor infiltrating immune cells and send out chemical signals for their immunosuppressive actions, rendering them weaken for attaking tumor. They also change the structure around the tumor, helping it grow, spread, and resist treatment. CAFs can even turn off immune cells by increasing immune “checkpoints” that block their function. Because of their major role in helping cancer thrive, understanding CAFs and how they communicate with immune cells is essential for improving cancer treatments, especially immunotherapy. This review highlights the latest knowledge about how CAFs shape an environment that protects tumors and resist tumor cell death.

## 1. Introduction

Genetic mutation and unrestrained growth of cancer cells was once thought to be an independent event for the progression of cancer. However, it is no longer recognized as an isolated event, seen instead as a complex biological drive orchestrating cellular interactions, growth factors, cytokines, and signaling pathways within the tumor microenvironment (TME) paving the way for tumorigenesis and metastasis. Tumor growth and metastasis are attributed to diverse dynamic changes that take place in the TME. Several studies in the past decades have explored the central and essential role of the TME in mediating the complex biological mechanisms of carcinogenesis [1]. The TME constitutes a complex ecosystem homing for various cell types including tumor cells, infiltrating immune cells, and extracellular matrix (ECM) [2,3]. The polarization and functional status of infiltrated immune cells are governed by the TME through modulating immune checkpoints on their surface and changing the tumor matrix surrounding the cells [3]. The TME has drawn much attention as potential target for cancer immunotherapy in recent years owing to its versatile role in tumor biology including immunosuppression [4,5,6,7].

Cancer-associated fibroblasts (CAFs) are the most abundant cells in the tumor stroma. They communicate with tumor cells via direct cell adhesion as well as indirectly by remodeling the extracellular matrix, which significantly influences the behavior of cancer cells. However, until recently, the biology of CAFs remained poorly defined because of the high heterogeneity in their origin and functional subtypes. It is generally agreed that CAFs are activated quiescent fibroblasts with significant heterogeneity and plasticity in their phenotypes and functions [8,9]. As they are overwhelmingly predominant in the stromal cell population, the immunoregulatory role of CAFs towards tumorigenesis, especially in solid tumors, is currently a subject of interest. A huge number of studies have already been carried out during the past decade, and many more are underway, to reveal the precise role of CAFs in the development of tumors and chemoresistance [10,11,12]. Evidence supports that CAFs can recruit and polarize many infiltrating immune cells towards tumorigenesis by secreting a number of cytokines and chemokines while simultaneously abrogating the migration of the most important cytotoxic CD8^+^ T cells at the tumor site [13]. Further, CAFs have been found to contribute directly to establishing an immunosuppressive TME and collaborate with other immune-inhibitory cells, including T regulatory cells (Treg), myeloid-derived suppressor cells (MDSCs), and M2-type macrophages, to enhance immune inhibition and immune evasion by tumor cells [14,15]. The immunosuppression loop in the TME is additionally accentuated by the release of various inflammatory cytokines, such as interleukin 1β (IL-1β), by activated immune cells that convert resident fibroblasts into proinflammatory CAFs, creating more immunosuppressive TME [10].

With the availability of immunotherapies for cancers, the treatment modalities have been dramatically transformed. Despite the impressive success shown in subsets of patients, the majority of cancer patients exhibit intrinsic or acquired resistance to these medicines [16,17,18]. Now, in many solid tumors, it has been shown that the prevalence of CAFs and the complexity of the TME have profound effects on the recruitment, infiltration, and cytotoxic action of T cells within the tumor [19]. Preclinical studies have demonstrated that activated CAFs are linked to tumor metastasis, resistance to treatments, disease recurrence, and a worse prognosis in many solid malignancies [20,21,22,23]. Immunomodulatory functions of CAFs affecting the efficacy of cancer immunotherapies have been cited in many recent investigations [24,25]. Thus, the potential of CAF-targeted cancer immunotherapies is becoming a fascinating area of research. To achieve success in CAF-targeted therapies and to overcome their role in chemoresistance, increasing understanding of the biology of CAFs and their crosstalk with cells in the TME are essential prerequisites.

## 2. Biology of CAF

Cancer-associated fibroblasts (CAFs) are indispensable sentinels of the TME developed through activation of resident fibroblasts under certain stromal conditions such as oxidative stress and local hypoxia. They play a pivotal role in tumor biology. Transdifferentiation of resident fibroblasts into CAFs also occurs through genetic alteration alongside the influence of stromal chemical factors. CAFs are the most important cellular elements in the tumor stroma and act as master players for the development and progress of tumors, including the development of therapeutic resistance. An immunosuppressive TME is greatly facilitated by CAFs through their orchestral interaction with tumors and infiltrating cells. CAFs contribute significantly to remodeling the ECM by increasing the deposition of matrix components, which triggers the fibrosis of intratumoral vessels and thus obstructs the infiltration of immune effector cells and the delivery of therapy to the tumor site. Antitumor activities of immune effector cells can be modulated by a large number of chemical factors released by CAFs, which include both cytokines and chemokines such as IL-6, TGF-β, FAP (fibroblast activation protein), SDF-1 (stromal-derived factor-1), VEGF (vascular endothelial growth factor), PGE2 (prostaglandin E2), CCL2 (C-C chemokine ligand 2), and IDO (indoleamine 2,3-dioxygenase). Various mechanisms and pathways are utilized by CAFs in achieving the target of an immunosuppressive TME, including (i) polarization of major immune cells into protumorigenic subsets; (ii) enhancing recruitment of inhibitory immune cells such as Treg, M2-TAMs (tumor-associated macrophages), N2-TANs (tumor-associated neutrophils), and rDCs (regulatory dendritic cells); and (iii) downregulating the cytotoxic activities of T cells and natural killer (NK) cells. Interestingly, because of their reciprocal relationship, CAFs can also be activated by all these cells, further enhancing the TME immunosuppressive loop. Additionally, many immune inhibitory checkpoints in the TME expression can be upregulated by CAFs, resulting in dysfunction of T cells and loss of immunosurveillance [26].

### 2.1. Origin of CAFs

**Figure 1:**
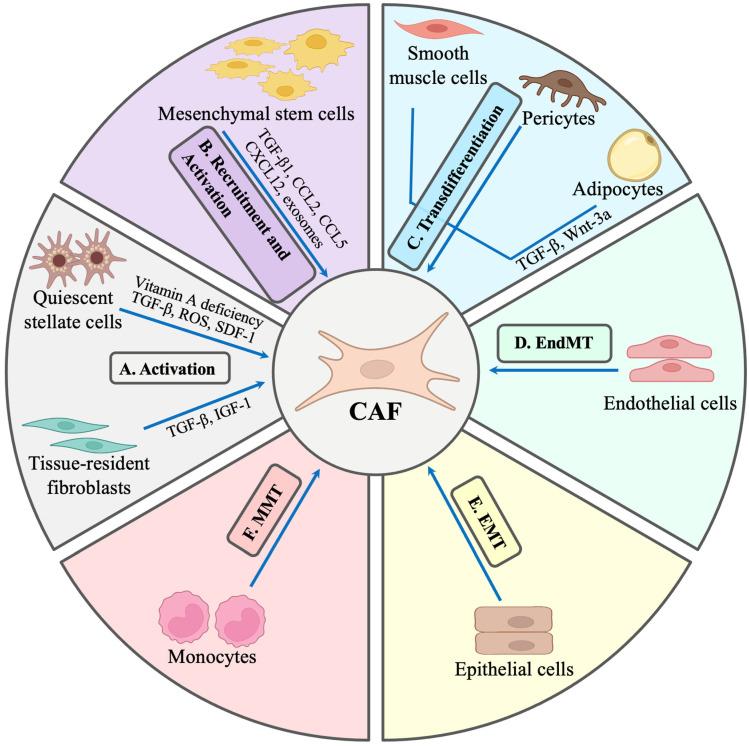
The cellular origins and related activation pathways of cancer-associated fibroblasts (CAFs): CAFs can be originated from multiple cell types by utilizing distinct mechanisms: A. ***Activation—***resident fibroblasts and quiescent stellate cells are converted into CAFs through stimulation by different modulators, such as TGF-β, HGF, PDGF, FGF-2, SDF-1, ROS, IGF-1 (insulin-like growth factor 1), and deficiency of vitamin A [37]; B. ***Recruitment and activation—***mesenchymal stem cells (MSCs) can be transdifferentiated into CAFs, induced by stimulating molecules such as TGF-β1, CCL2, CCL5, CXCL12, and tumor-derived exosomes [38]; C. ***Transdifferentiation—***adipocytes, pericytes, and smooth muscle cells can be transdifferentiated into CAFs by TGF-β1 and Wnt3a [39]; D. ***EndMT—***endothelial cells are transformed into CAFs through endothelial–mesenchymal transition [40]; E. ***EMT—***epithelial cells are transformed into CAFs through epithelial–mesenchymal transition [41]; F. ***MMT—***monocytes are transformed into CAFs through monocyte–myofibroblast transdifferentiation [42] (created with BioRender.com icons).

The sources of origin and subpopulations of CAFs remain ambiguous because of the lack of discernible biomarkers for the lineage, which has led to high plasticity in their phenotypic and functional diversity. Because of the great heterogeneity in their origins, the exact morphology of CAFs is difficult to describe. Nevertheless, they are large, contractile, fusiform mesenchymal cells with indented nuclei and branching cytoplasm. Considering their extreme heterogeneity, high plasticity, and extensive variation within subpopulations, CAFs better represent a state of activated fibroblasts than a specific cell archetype. Although this is not sufficient to exactly distinguish them from the normal counterparts, CAFs have certain unique cell surface markers and morphological attributes for their identification. With regards to cellular status, sources, and functions, CAFs are quite heterogeneous. Originally, it was thought that CAFs were transformed fibroblasts, but based on their sources and defined characteristics, it was noted that transformation of many classes of cell, such as adipocytes, pericytes, epithelial cells, endothelial cells, smooth muscle cells, and mesenchymal stem cells, also leads to the generation of CAFs (Figure 1) [27,28,29,30,31,32]. For simplicity, CAFs are defined as highly versatile, plastic, and resilient stromal cells that are different from tumor, epithelial, endothelial, and immune cells. According to a large number of studies, mesenchymal stem cells (MSCs) show a homing tendency towards tumor stroma, and bone marrow mesenchymal stem cells (BMSCs) have been found to be the major precursors of CAF [27,33]. Under certain conditions and cytokine stimulation, a variety of cell types, including epithelial cells, endothelial cells, and CAFs, can originate through differentiation of multipotent BMSCs. A process called epithelial–mesenchymal transition (EMT) allows the transformation of a polarized epithelial cell to a mesenchymal phenotype from BMSCs under the influence of cytokines and growth factors. The transformed cells lose their cell polarity and tight junction and consequently become migratory and invasive in nature [34,35,36].

It has been demonstrated that adipocytes, especially white adipocytes, also constitute CAF precursors. In breast cancers, under the influence of tumor-induced transforming growth factor β1 (TGFβ1), adipose tissue-derived mesenchymal stem cells (ADSCs) have been transdifferentiated into CAF phenotypes [43,44,45]. Moreover, it has been shown that breast-cancer-derived Wnt3a (a member of *Wnt* gene family, clustered on human chromosome 1q42) can also generate adipocyte-derived fibroblasts (ADFs) through differentiation of adipocytes that express high levels of a CAF-specific marker called fibroblast specific protein 1 (FSP-1) through a highly conserved signal transduction pathway called the Wnt/β-catenin pathway [10,46]. Additionally, endothelial cells can also be transdifferentiated through endothelial–mesenchymal transition (EndMT) and give rise to CAFs under the influence of TGFβ1. Transdifferentiation of epithelial and endothelial cells into CAFs through EMT and EndMT, respectively, enables them to express fibroblast-specific markers such as FAP and α SMA (α smooth muscle actin) [47]. Meanwhile, peritoneal mesothelial cells (epithelial monolayer lining the cavities) can also be differentiated into CAFs via mesothelial–mesenchymal transition (MMT) induced by TGFβ1 [48]. Further, in pancreatic ductal adenocarcinoma, a special type of CAFs called myofibroblasts has been generated through monocyte–myofibroblast transdifferentiation (MMT) utilizing an oxidative-stress-dependent p38-mitogen-activated protein (MAP) kinase signaling pathway [49]. Furthermore, pancreatic stellate cells (PSCs) and hepatic stellate cells (HSCs) have been found to be the sources of CAFs with expression of surface αSMA marker upon activation by TGFβ and platelet-derived growth factor (PDGF) [29,50]. The activation and differentiation of all these cells into CAF phenotypes are mediated by different tumor-cell-derived factors utilizing diverse mechanisms and signaling pathways [29]. Thus, essentially, the cellular origins of CAFs are ambiguous and heterogenous in different tumor types, different subtypes may have divergent origins, and even within a specific tumor, populations of subtypes may have varied origins and functional phenotypes.

### 2.2. Activators of CAFs

Recruitment and activation of quiescent tissue-resident fibroblasts into CAFs involves a variety of tumor-cell-derived activators and modulators, including TGFβ, fibroblast growth factor 2 (FGF-2), hepatocyte growth factor (HGF), PDGF, stromal-derived factor-1 (SDF-1) and signaling pathways [37] (Figure 1). Hypoxia, induced by the accumulation of reactive oxygen species (ROS), is a typical feature of the tumor microenvironment that arises because of tumor vascular dysfunction. Large amounts of oxygen being consumed by the rapidly proliferating tumor cells is the main cause of the hypoxic microenvironment, which results in alteration of the expression levels of genes that regulate metabolism and other processes, making intercellular communication more complex and shaping a tumor-promoting and metastatic TME [51].

Sources and categories of activator and signaling pathways vary widely across different tumor types. Among several internal signaling pathways, the role of TGFβ in the conversion of MSCs into CAFs has long been established. Further, the recruitment and transformation of MSCs are further aided by TME-secreted angiogenic motifs such as C-C chemokine ligands (CCL), e.g., CCL2, CCL5, and C-X-C motif chemokine ligand 12 (CXCL12) [52]. For example, in prostate cancer, both tumor- and stromal-cell-induced TGFβ1 act as activators in the transformation of MSCs into CAFs [53]. In breast cancer, transdifferentiation of BMSCs into distinct CAF subsets is mediated by myeloid zinc finger 1 (MZF1)-induced TGFβ1 through the osteopontin–TGF-β1 pathway [54].

The promotion and activation of CAFs are stimulated by various TME-released factors using a particular internal signaling pathway. The activation of the Janus kinase/TNF inducer and activator of transcription 3 (JAK/STAT3) and nuclear factor-kappa B (NF-κB) signaling pathways is involved in accelerating the malignant potential of CAFs through the release of proinflammatory mediators such as IL-1β, IL-2, IL-6 and TNF-α [46]. It has been observed that CAF-induced inflammasomes are important regulators of cancer development and control. Upon recognition of the damage-associated molecular patterns (DAMPs) in cancer cells, CAF-mediated aberrant activation of inflammasomes takes place via NLRP3 inflammasome signaling pathway. Activated inflammasomes release IL-1β in turn to elicit inflammation through oligomerization and activation of inflammatory caspases [55]. Additionally, it has been demonstrated that Dickkopf-3 (Dkk3, a heat shock factor 1 effector)-driven β-catenin and the YAP/TAZ signaling pathway can activate CAFs that behave aggressively in promoting tumor phenotypes [52]. It is worth noting that several epigenetic modifications such as acetylation, methylation, and phosphorylation induced by the JAK1/STAT3 signaling pathway in normal fibroblasts are required along with proinflammatory cytokines to initiate and maintain a proinvasive CAF phenotype [56]. Proinflammatory cytokines such as leukemia inhibitory factor (LIF) and IL-6, released by both cancer cells and CAFs, contribute to the epigenetic modification of CAFs, leading to enhanced protumorigenic function by remodeling of the extracellular matrix. Further, CAF activation can also be mediated via hypoxic tumor microenvironment and matrix stiffening [57]. In summary, several tumor-induced chemical factors can activate CAFs utilizing multiple signaling pathways, and activated CAFs in turn exhibit their protumorigenic actions through remodeling of the ECM, promotion of angiogenesis, invasion, metastasis, immunosuppression, and even chemoresistance.

### 2.3. Markers and Functional Phenotypes of CAFs

**Figure 2:**
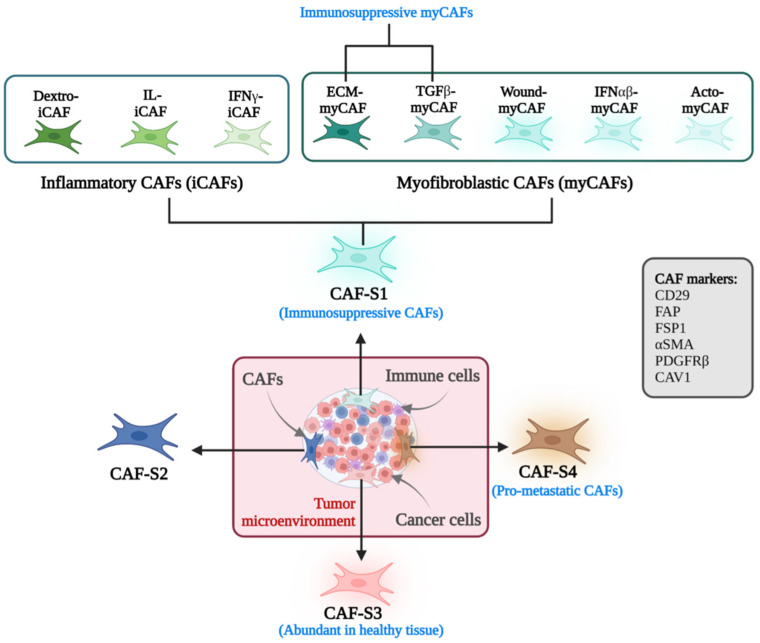
Schematic representation of CAF subpopulations. CAFs represent a highly plastic cellular population within the TME. Four different CAFs populations (CAF-S1 to CAF-S4) were identified based on analysis of six CAF markers. CAF-S1 presents an immunosuppressive function, while CAF-S4 promotes tumor metastasis. Based on single-cell analysis, CAF-S1 exhibits 2 major subpopulations, myofibroblastic (myCAF) and inflammatory (iCAF), with 5 clusters belonging to the myCAFs and 3 clusters belonging to the iCAFs. Both ecm-myCAF and TGFß-myCAF exhibit immunosuppressive functions (Created in BioRender. Amin, M. (2025) https://BioRender.com/dtwg60v (accessed on 16 June 2025)).

Cancer-associated fibroblasts are perpetually activated fibroblasts, and their identification typically relies on the expression of several CAF markers. However, as mentioned above, because of the degeneracy of markers, a precise definition and CAF-specific marker are yet to be assigned for accurate description of CAFs. Nonetheless, high expression of markers such as αSMA, PDGFRα, and FAP has been widely used to separate CAFs from the larger pool of fibroblasts. The heterogeneity of CAF markers is tumor-dependent and varies strongly between different CAF subpopulations across tumors [58]. Moreover, overlapping in expression of some of these markers in other mesenchymal cell types poses a consequential challenge in defining the biological role of CAFs in cancers [57]. Until recently, CAFs in the TME were best characterized by their functional phenotypes rather than biological markers because of a lack of consensus. Similarly to their heterogenous cellular origins and existence, CAFs exhibit many subpopulations with diverse putative functions. It has long been understood that based on their functional status, there are at least two major CAFs, quiescent and activated, differentiated by their own somewhat unique markers. Under homeostatic settings and metabolic status, quiescent CAFs resemble normal fibroblasts with low proliferative ability [25], while activated CAFs, also known as myofibroblasts, have increased proliferative capacity, metabolic activity, and production of extracellular matrix (ECM). Although activated fibroblasts are intended for a “wound healing” process to maintain tissue equilibrium, they are taken over by tumor cells for their development and survival, including metastasis [25,59,60]. Regarding markers, vimentin (VIM) is unique to quiescent CAFs, while others, such as FSP-1, PDGFR-α/PDGFR-β, α-SMA, FAP, integrin β1 (CD29), periostin (POSTN), and PDPN, have been discovered to better characterize activated CAFs [61,62]. Although several of these markers identify certain CAF subpopulations with distinctive expression profiles, a degree of overlapping indicates their heterogeneity in the TME [63]. In a concurrent study of six CAF markers, viz., α-SMA, FAP, FSP-1, CD29, PDGFR-β, and CAV-1 (*caveolin-1*), four distinct CAF subpopulations, viz., CAF-S1, S2, S3, and S4, were found in human breast and ovarian cancers with distinct expression profiles (Figure 2) [64,65]. Interestingly, quiescent fibroblasts present in tissues outside tumors had similar characteristics to CAF-S2 and CAF-S3 (FAP^−^ CD29^low-med^ SMA^−^). On the contrary, myofibroblasts, found exclusively in tumors and metastatic lymph nodes, showed CAF-S1 (FAP^high^ CD29^med-high^ SMA^high^) and CAF-S4 (FAP^−^SMA^high^ CD29^high^) phenotypes. Further, CAF-S1 exhibited ECM adhesion and wound-healing signature along with immunosuppressive action due to a marked increase in number of Treg cells (CD4^+^ CD25^+^ FOXP3^+^), in contrast to the perivascular contraction signature in CAF-S4, which encouraged metastasis. Studies have confirmed the presence of both ECM-rich CAF-S1 and contraction-rich CAF-S4 subpopulations in various cancer types, including pancreatic, colorectal, breast, lung, and head and neck, in both human and mouse cancer models [66,67,68,69,70,71]. Several preclinical studies in pancreatic, ovarian, and lung cancers have discovered two distinct subpopulations of CAFs, known as myofibroblast CAFs (myCAFs) and inflammatory CAFs (iCAFs), at different locations in tumors [72,73]. Located primarily around the tumor bulk, myCAFs expressed strong α-SMA with a highly matrix-producing, contractile phenotype, while iCAFs were more remote from the tumor margins, in the desmoplastic area of the tumor, and characterized by the expression of an immunomodulatory secretome including IL-6 and CXCL12 [12,74]. Further, transcriptome study demonstrated that myCAFs and iCAFs were driven by different signaling pathways; for example, TGFβ and IL-1 were associated with myCAFs and iCAFs, respectively [66]. In conjunction with subpopulations of CAFs, it is worth mentioning that eight distinct clusters of CAFs were found in breast cancer in a recent single-cell investigation. Of eight clusters, three (1, 2, and 5) belonged to the iCAF subgroup and were driven by different signals/pathways, the detox pathway, IL-signaling, and the IFNγ-related pathway, respectively. The myCAF subgroup contained the other five clusters (0, 3, 4, 6, and 7), which were related to ECM proteins, the TGFβ-dependent pathway, wound healing signaling, the IFNαβ-related pathway, and actomyosin signaling, respectively (Figure 2) [71]. Besides the classical myCAFs and iCAFs, a third subset of CAFs called antigen-presenting CAFs (apCAF) was recently discovered in pancreatic ductal adenocarcinoma (PDA) through multiple single-cell RNA sequencing (scRNA-seq) studies. This subset was found within the IFNγ–iCAF cluster, characterized by highly expressed MHC class II and CD74 protein and able to present antigens to T cells in vitro. However, further validation is required regarding the locations and activation conditions of apCAF [74].

In summary, it is quite evident that CAFs have heterogenous cells of origin and activators that contribute to their highly pleiotropic role in tumor biology. Further, different CAF subpopulations are involved in different tumor entities because of their high heterogeneity and plasticity. As evidenced by several lines of investigation, protumorigenic functions of CAFs are largely attributable to their ability to reprogram and reshape the tumor metabolic microenvironment [75,76].

## 3. Tumor Microenvironment and Crosstalk Between CAFs and Tumor-Infiltrating Immune Cells

**Figure 3:**
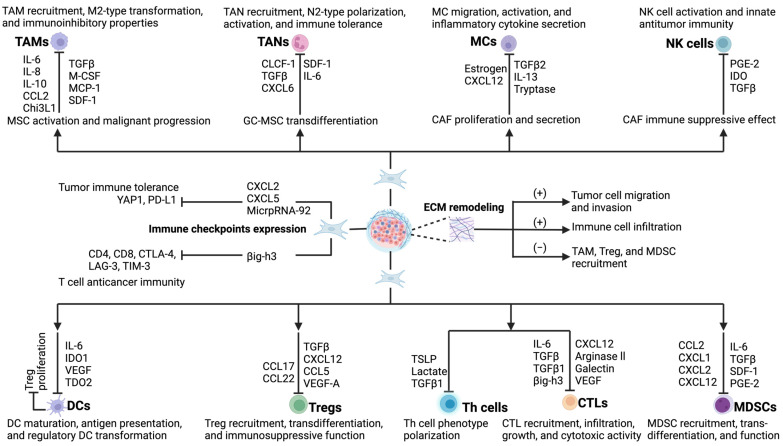
CAF-mediated recruitment of immune cells and mechanisms of immunosuppression in the TME. CAFs shape the tumor microenvironment toward a protumorigenic and immunosuppressive milieu by recruiting and affecting functions of various innate and adaptive immune cells. CAFs modulate the antitumor activity of immune cells through the secretion of multiple chemokines, cytokines, and other effector molecules. They (i) promote the transdifferentiation or polarization of immune cells such as tumor-associated macrophages (TAMs), tumor-associated neutrophils (TANs), mast cells (MCs), dendritic cells (DCs), and T lymphocytes into certain protumorigenic cell subsets; (ii) recruit and facilitate the activities of immune-inhibitory cells, including M2-type TAMs, N2-type TANs, regulatory DCs (rDCs), regulatory T (Treg) cells, and myeloid-derived suppressor cells (MDSCs); and (iii) restrict the cytotoxic activity and cytokine production of NK cells and cytotoxic T lymphocytes (CTLs). Reciprocally, several infiltrating immune cells such as TAMs, TANs, MCs, and DCs can contribute to the formation of immune-suppressive loops by effecting CAF activation and function. CAFs can also upregulate the expression of immune checkpoint molecules such as PD-1/PD-L1, CTLA4)/B7, LAG-3, and TIM-3 to induce T-cell (CD8 and CD4) dysfunction. Moreover, CAF-mediated remodeling of the ECM facilitates immune suppression through the production of fibronectin, collagen, MMPs, and activation of the focal adhesion kinase (FAK) signaling pathway (Created in BioRender. Amin, M. (2025) https://BioRender.com/92cbdud (accessed on 16 June 2025)).

The ecosystem surrounding a developing tumor is called the tumor microenvironment. It is a complex and unique environment created by the interactions of tumor cells with the host tissue and remains a seemingly ever-increasing entity in the progression of tumor including metastasis. TME is under constant evolution that homes for numerous cellular elements, including tumor and stromal cells (e.g., fibroblasts, endothelial cells, adipocytes, and inflammatory and immune cells). Besides cellular components, various noncellular elements such as the extracellular matrix (ECM), arrays of cytokines and chemokines, specialized conditions such as hypoxia (induced by the activation of hypoxia-responsive genes in tumor cells), and interstitial pressure are integral parts of a TME. A highly selective milieu is created in the TME by the continuous evolution in molecular and cellular events dominated by the tumor cells. The ECM is a dynamic protein scaffold of crosslinked macromolecules, such as collagens, fibrous proteins, glycoproteins, proteoglycans, and polysaccharides, that provides architectural and anchorage support to all cellular components in TME. Further, the ECM acts as the niche for structural and biochemical support of pluripotent cancer stem cells (CSCs) and regulates a number of their integral processes, including proliferation, self-renewal, homeostasis, morphogenesis, and differentiation through various growth factors and intracellular signaling pathways [77]. All these cells and noncellular components contribute to the wider microenvironment of tumor, but among them all, infiltrating fibroblasts are suggested to be the principal player in tumorigenesis at all stages through their enormous ECM-remodeling capacity. During tumorigenesis, the hypoxic TME and tumor cells secrete chemokines, cytokines, and growth factors, especially TGFβ, which activates quiescent fibroblasts and transforms them into CAFs that constitute the overwhelming cell population in the ECM. The emergence of CAFs greatly influences the tumor microenvironment by releasing a plethora of cytokines, chemokines, growth factors, and exosomes such as VEGFA and CXCL12 to reciprocally complement the tumor growth. CAFs are a highly heterogeneous cell population with diverse functions such as enhancing the migration of other cells into the TME, altering tumor cell metabolism, polarizing immune cells to anticancer response, and contributing to drug resistance [63]. As part of defense, distinct immune cell populations infiltrate into the TME to show their antitumor activities in the first place, but with progression, the TME seriously impedes the functions of these immune cells. TME in general and CAFs in particular polarize immune cells, viz., regulatory T cells (Tregs), M2 macrophages, N2 neutrophils, and regulatory dendritic cells (rDCs), towards tumorigenesis by actively downregulating their antitumor activities through different mechanisms and signaling pathways. The antitumor potential of immune cells is greatly hindered by CAF-induced cytokines and chemokines, even beyond their creating an immunosuppressive TME to enable cancer cells to evade immune surveillance [14,78]. The following section briefly describes the interaction of CAFs and major immune effector cells in promoting cancer (Figure 3).

### 3.1. Interaction of CAFs and Tumor-Associated Macrophages (TAMs)

Macrophages are prodigious immunocompetent cells that play vital roles in immunity through engulfing and digesting foreign substances and bridging between innate and adaptive immune responses. They are highly plastic innate immune cells of myeloid origin, sentinels that maintain equilibrium in tissue and possess antitumor activities. The differentiation of macrophages into distinct functional phenotypes is governed by a myriad of environmental cues, driving their evolution towards either the M1 phenotype, characterized by a proinflammatory response initiated in a classical manner, or the M2 phenotype, indicative of a traditionally elicited immunoregulatory response. The transition to the M1 or M2 state encompasses a complex regulatory framework, entailing an array of signaling cascades, transcriptional and epigenetic mechanisms, and post-transcriptional regulatory dynamics [79]. TAMs can differentiate from circulating monocytes that infiltrate tumors via the blood vessels but may also derive from tissue-resident macrophages (TRMs) that infiltrate tumors directly from the surrounding tissues [80]. Approximately half of stroma-infiltrating immune cells are TAMs, which display considerable phenotypic plasticity but play a significant role in the progression of tumors. Different polarizing cytokines released by Th1 and Th2 are responsible for activating M1-type and M2-type TAMs, respectively. Proinflammatory or M1-TAMs are activated mainly by bacterial lipopolysaccharide (LPS), IL-1β, and IFN-γ (secreted by Th1 cells, cytotoxic T cells, and NK cells, respectively), while Th2-released cytokines such as IL-4, IL-13, CSF-1, IL-10, and TGF-β activate M2-TAMs [81]. Upon activation, high levels of class II MHC molecules are expressed on M1-TAMs, and they play an antitumor role early in tumorigenesis by releasing an array of proinflammatory cytokines such as IL-1, IL-6, TNF, ROS, and nitric oxide [82]. On the contrary, M2-TAMs create an immunosuppressive TME and favor tumor cells for invasion, metastasis, angiogenesis, and remodeling of the ECM by secreting high levels of anti-inflammatory cytokines such as interleukin IL-4, IL-13, and IL-10 [83]. Besides cytokine-induced polarization, hypoxia and lactate can also polarize macrophages into M2 type through noncytokine extrinsic polarization pathways within TME.

A large number of cytokines, chemokines, and growth factors secreted by M2-TAMs, including TGF-β, PDGF, IL-6, IL-10, CXCL, COX-2, VEGF, and PDGF, favor angiogenesis and tumor progression. Similar to CAFs, M2-TAMs are very efficient contributors to an immunosuppressive tumor microenvironment through limiting the activity of tumor-infiltrating T cells and NK cells and increasing the population of regulatory T cells (Treg). The development and progression of both primary and metastatic tumors can be influenced by TAMs. For example, angiogenesis in the primary tumor facilitates the invasion and modulation of premetastatic sites, aiding in tumor dissemination. Further, M2-TAMs are involved in the development of therapeutic resistance in tumors by different mechanisms, including activation of STAT3 in tumor cells, enhancing EMT, and upregulating checkpoint inhibitors [84].

For supportive roles in tumor growth and progression, both CAFs and TAMs are among the most important and abundant players of the tumor microenvironment. There exists a dynamic and mutual interrelationship between these two cell populations; they influence each other reciprocally and crosstalk with tumor cells. TAMs always remain in the immediate proximity of CAF-rich areas in the TME, indicating their intimate interactions and joint cooperation in inducing and modulating an immunosuppressive TME [85]. Recruitment of macrophages and other immune cells is under the purview of the regulatory functions of CAFs in addition to their functional alteration of the TME. There is abundant evidence that CAFs can recruit cells of mononuclear phagocyte systems via a variety of regulatory molecules and transform them into the protumorigenic TAM subset (M2-TAM) [86]. In breast cancer, it was found that the migration and polarization of monocytes into M2-TAMs was promoted by CAF-secreted monocyte chemotactic protein-1 (MCP-1), IL-6, CXCL12, and chitinase 3-like protein 1 (Chi3L1) [87]. Similarly, in prostate cancer, monocyte recruitment and polarization into the M2 phenotype was aided by CAF-secreted SDF-1 and CXCL14 [88]. It has also been shown that the migration and differentiation of bone-marrow-derived monocytes into M2 macrophages can be facilitated by other cytokines secreted by CAFs, such as GM-CSF, IL-8, IL-10, TGFβ, and CCL2 (seen in skin tumors) [89]. Moreover, CAF-induced M2-TAMs were found to have higher expression of programmed cell death protein 1 (PD-1) on their cell surfaces [87], not only reducing their phagocytic activity against tumor cells but impeding their role in proliferating cytotoxic T cells [90].

In turn, M2-phenotype TAMs can induce the activation and development of CAFs via a variety of mechanisms, including acceleration of epithelial–mesothelial transition via paracrine production of TGF-β1. Macrophage-derived factors such as granulin (a secreted glycoprotein) have been found to activate and transform resident hepatic stellate cells into myofibroblasts, resulting in fibrosis, as seen in pancreatic ductal adenocarcinoma metastasis to the liver [91]. TAMs have been recently demonstrated to have an impact on MSC activity and the transdifferentiation of CAFs, with enhanced transformation of gastric epithelial cells into cancer cells [92]. Further, in an in vitro study of the progression of neuroblastoma, it was observed that TAM-like macrophages derived from peripheral blood mononuclear cells (PBMCs) were able to promote the proliferation and invasiveness of CAF-like BM-MSCs [93]. Since both CAFs and TAMs can reciprocate each other’s activation and functions, they share many markers in common. Poor clinical prognosis in many solid tumors has been observed with high expression of markers common to both CAFs and TAMs, including αSMA, FAP, CD163, and CD209 [94].

In conclusion, both CAFs and TAMs exert a plethora of effects on tumor progression, metastasis, and the immunosuppression loop, and together, they have synergistic pleiotropic effects on the tumor cells [95]. Although both CAFs and TAMs are critical stromal cells in solid tumors, details about their mutual relationship and roles in cancers are yet to be elucidated. Thus, further studies are recommended for better understanding the biology, reciprocal interaction, and plasticity in the functions of both CAFs and TAMs in the TME, with an eventual goal of designing targeted therapies against these tumor master players.

### 3.2. Interaction of CAFs and Tumor-Associated Neutrophils (TANs)

Neutrophils are the most abundant granulocytes; they constitute 40–70% of the white blood cells in human blood. They play a very important role in inflammation and act as first line phagocytic cells against invading pathogens. It was believed until recently that, being short-lived cells, neutrophils are merely silent bystanders in the tumor microenvironment. However, expanding research over the past decade has now established that neutrophils are important contributing cells in the development and progression of cancer. The neutrophil-to-lymphocyte ratio (NLR) has been used as a valid indicator of the prognosis of solid tumors, and a poor clinical outcome in many cancers is correlated with a high NLR. Notably, a significant part of the TME is formed by infiltrating neutrophils, called tumor-associated neutrophils (TANs), which are characterized by significant phenotypic diversity and functional resilience. TANs constitute a highly divergent cell population that have tumor-inhibiting and -promoting functions. Neutrophils in the bone marrow are recruited and polarized by several TME-derived factors, and in return, polarized neutrophils interact with other stromal cells, in particular immune cells in the TME, via secreted chemical mediators for tumor progression. Like macrophages, following stimulation by tumor-derived cytokines including IL-1β, IFN-β, TGF-β, IL-8, and prostaglandin E2 (PGE2), TANs acquire the potential to be polarized to either the antitumor N1 (mainly IFN-β induced) or protumor N2 (mainly TGF-β induced) phenotype (Figure 3). Generally, N1 TANs show a mature phenotype with highly cytotoxic and immunoactivating ability. In contrast, the N2 TANs are a long-lived immature phenotype and show low cytotoxicity but high tumorigenic and immunosuppressive activity. The antitumor functions of N1 TANs are attributed to increased production of reactive oxygen species (ROS), neutrophil extracellular traps (NETs), intercellular adhesion molecule 1 (ICAM1), and tumor necrosis factor-α (TNF-α), while the protumor activities of N2 TANs are associated with different cytokine profiles including increased expression of VEGF, MMP-9, and CXCR4. It has been observed that neutrophils are recruited and polarized to TANs by the TME, imparting them with the potential to regulate the growth and progress of tumors, similarly to TAMs. The antitumor effects of N1 TANs are executed by direct cellular killing through ADCC (antibody dependent cellular cytotoxicity) or indirectly via activation of the antitumor activities of other immune cells. Additionally, cytotoxic effects on tumor cells are further aided by N1 TANs through the production of neutrophil elastase (NE), ROS, and NO and Fas-FasL-mediated apoptosis. On the contrary, in coexistence with M2-TAMs, N2 TANs display protumorigenic roles through several processes, such as cellular migration, angiogenesis, proliferation, NETs, and the formation of a premetastatic niche. Additionally, by secreting IL-8, TNF-α, and myeloperoxidase (MPO), N2 TANs can activate macrophages and establish the TAN–TAM axis, which has been observed in several cancer types [96]. Moreover, by secreting ECM-remodeling enzymes, N2 TANs can regulate immunosuppression that facilitates tumor progression. Unlike TAMs, there is a lack of strong evidence of polarizing molecules for TANs. Differences in degrees of activation may lead to N1 or N2 TAN phenotypes [97].

CAFs modulate the biological properties of tumors and tumor-infiltrating immune cells, including neutrophils, in multiple ways. Various bioactive molecules released by CAFs have effects, through paracrine and autocrine signaling, on the direct or indirect polarization of TANs towards the protumor phenotype. CAFs are able to recruit neutrophils from the peripheral blood to the tumor microenvironment by expressing CXCR2 and secreting SDF-1α [14] and induce TANs to contribute to the development of an immunosuppressive TME [98]. In a recent study on hepatocellular carcinoma (HCC), indirect polarization of TAN to the N2 phenotype was observed by the CAF-induced cytokine factor 1 (CF1), which upregulated the expression of TGF-β and CXCL6 on tumor cells [99]. Further, through an IL6–STAT3–PDL1 signaling cascade, HCC-CAFs were found to regulate the survival, activation, and function of neutrophils within the microenvironment of HCC [100]. Studies have revealed that TAN-secreted IL-17, IL-23, and TNF-α and activation of the protein kinase B/p38 (Akt/p38) pathway can reciprocally induce the transformation of mesenchymal stem cells into CAFs [101].

### 3.3. Interaction of CAFs and Mast Cells (MCs)

Mast cells (MCs) are terminally differentiated immune cells of the myeloid lineage found as residents in the connective tissue. Upon activation through appropriate signals, MCs mediate defense against a wide range of pathogens through degranulation of vasoactive amines. Long-lived secretory sentinels, MCs are able to rapidly respond to modifications in the environment. Apart from their protective role, MCs are also involved in allergies and cancers through activation of specific signaling pathways. Tumor-associated MCs (TAMCs) are infiltrating cells within the TME that can play a role in the inhibition or promotion of tumorigenesis. Differences in the responses of mast cells are attributable to their ability to release different cytokine profiles, either proinflammatory (e.g., TNF-α, IL-1 β, IL-6, IFN-γ) or anti-inflammatory (e.g., IL-4, IL-10, IL-13, TGF-β). As important sentinels between innate and adaptive immunity, infiltrating MCs are contended immune cells in the TME because of their dual role. They initially exhibit antitumor functions through recruitment of other immune effector cells via the release of an array of proinflammatory mediators but later play a tumor-promoting role by augmenting angiogenesis, lymphangiogenesis, fibrosis, and metastasis. This dichotomy in the role of TAMCs relies on their capacity to secrete a broad spectrum of regulatory molecules, which largely depends on the tumor type and grade. In their capacity as cancer promoters, TAMCs strongly facilitate angiogenesis through the liberation of a number of angiogenic molecules, e.g., TGF-β, EGF, fibroblast growth factor-2 (FGF-2), stem cell factor (SCF), heparin, histamine, MMP-9, and proteases [102]. On the contrary, various chemical mediators released by TAMCs, such as TNF-α, IL-1, IL-6, tryptase, chondroitin, and sulfate, trigger antitumor immune responses, including inflammation leading to apoptosis in the tumor [103].

Like other immune cells, CAFs and TAMCs have an interdependent and reciprocal relationship. Increased numbers of CAFs and TAMCs present in any tumor islets correlate directly to the tumor progression and aggressiveness of a cancer [104]. In prostate cancer with estrogen overexpression, recruitment and proliferation of TAMCs with an inflammatory cytokine profile was mediated by CAF-derived CXCL12, leading to a protumorigenic effect [105]. Conversely, the same study also discovered that TAMCs have a stimulatory effect on CAFs through the release of tryptase and IL-13, which can promote CAF proliferation without the aid of the TGFβ2–STAT6 signaling pathway [106]. Further, it has been noted that TAMCs in neurofibromas can prompt the process of transdifferentiation of fibroblasts into myofibroblasts, which subsequently increases the tumorigenic activity of TAMCs through the TGFβ signaling pathway [107]. Cell–cell cooperation between CAFs and TAMCs was recently explored in human prostate cancer via an in vitro microtissue model to demonstrate the transition of benign epithelial cells to an early malignant morphology [108]. Thus, it is evident that together TAMCs and CAFs contribute significantly to the development and progress of tumors, and targeting mediators released by TAMCs holds novel promise in targeted cancer therapeutics.

### 3.4. Interaction of CAFs and Natural Killer (NK) Cells

Originating from a common progenitor, NK cells belong to the same family as B and T lymphocytes. Morphologically, they are large, granular cytotoxic cells that function as important defense cells at the interface of innate and adaptive immunity. NK cells are vigilant early in host defense against physiologically stressed cells such as tumor cells and virus-infected cells through their unique activation mechanisms. Like T cells, they lack specific receptors such as CD3 or T cell receptors (TCRs) but are capable of performing contact-dependent cytotoxicity to tumor cells as well as immune modulation by cytokine production, similarly to cytotoxic T cells. For surface markers, NK cells are CD3^−^CD56^+^ and can be further divided into the CD56^bright^ (similar to Th cells, regulating immune response) and CD56^dim^ (mainly cytotoxic) subgroups. In absence of specific receptors for discriminating capricious antigens, NK cells possess general activation and inhibitory receptors. Activation and cytotoxic effects are directed towards target cells that do not display antigens with self MHC class I molecules, called activation by “missing self”. The stimulation and effector functions of NK cells can be mediated upon integration of signals derived from recognition of NKG2D ligands on the target cells, called an “induced self” signal [109]. The activating receptors of NK cells fall under at least three categories based on the corresponding ligands, viz., MHC-class-I-specific (e.g., Ly49c, Ly49i, NKG2C, NKG2E, NKp80 etc.), MHC-I-related (e.g., NKG2D), and MHC-class-I-nonrelated (e.g., CD16, α4 integrin, DNAM-1, NKp46, NKp30, NKp44 etc.) receptors, while the main inhibitory receptors include Ly49s, NKG2A, and LLT1 [110]. Moreover, several cytokines such as IL-2, IL-12, IL-15, IL-18, and IFN-α and IFN-β (type I interferons) can activate NK cells, while IL-6, IL-10, and TGF-β suppress the activity of NK cells. Activation or inhibition of NK cells is primarily dependent on the expression of MHC I molecules by the target cells. Nonmalignant target cells such as virus-infected cells express high levels of MHC class I molecules and are recognized by the inhibitory receptors of NK cells, while cancer cells can become sensitive to NK cell lysis because of their very low expression of MHC class I molecules that signal the activation of NK cells [111].

The cytotoxic functions of tumor-infiltrating NK cells can be impaired by various soluble inhibitory factors released by CAFs in the TME of solid tumors [112]. Numerous studies have suggested that CAF-induced inhibitory factors can affect NK receptor activation, cytotoxic activity, and cytokine production through a number of direct and indirect mechanisms [112]. Multiple activators and effector molecules may be involved in the interaction of CAFs and NK cells, the precise mechanism of which is complex and yet to be explored. According to numerous reports, CAF-secreted TGFβ is the most powerful cytokine that connects CAFs and NK cells in the TME and significantly reduces the activation and cytotoxic activity of NK cells [78]. The potential mechanism by which CAFs negatively regulate NK cells is thought to be the downregulation of their activation receptors, such as NKG2D, by the action of TGFβ, which in turn causes NK cells to produce decreased amounts of interferons [113]. In several cancer types, the CAF-activated SMAD2/3-dependent signaling pathway has been found to be responsible for in vitro selective reduction of the expression of MHC-class-I-nonrelated activating receptors, such as NKp30, NKp46, NKG2D, and DNAM-1, in NK cells [112]. For instance, expression of NKp30, NKp44, and DNAM-1 and release of cytolytic granules by NK cells were found to be inhibited by CAF-released PGE2 in melanoma [24], while in hepatocellular carcinoma, CAF-induced PGE2 and IDO were found to be involved in transdifferentiating NK cells into an inactivated phenotype, rendering them to an unresponsive antitumor state [114]. Additionally, CAFs can inhibit the secretion of cytokines such as TNF-α and IFN-γ and cytotoxic granules containing perforin and granulase B by NK cells, suggesting tumor-promoting effects of CAFs on NK cells. Further, CAF-polarized stromal cells such as macrophages, DCs, and Treg cells also extend their inhibitory effects to NK cells to jeopardize the cytotoxic activity against tumor cells [115]. It is interesting to learn that NK cells themselves can help CAFs to form the immunosuppressive loop in the TME by encouraging PGE2 secretion [76]. In a preclinical study, when cocultured with NK cells, CAFs were found to express higher PGE2 than normal fibroblasts, indicating influence of NK cells in the expression of this specific protein by the CAFs [76]. Although the exact intracellular mechanisms involved in the suppression axis of NK cells’ functions are not fully elucidated yet, TGFβ-mediated suppression of NKG2D activation receptors is thought to play an important role. More studies are required to better understand the biology of CAFs and NK cells and to unravel the underlying molecular and cellular mechanisms in their crosstalk. With further knowledge, the landscape of cancer treatment can be modified by designing NK-cell-based immunotherapeutics.

### 3.5. Interaction of CAFs and Dendritic Cells (DCs)

Dendritic cells (DCs) are a unique population of hemopoietic cells likely to derive from myeloid precursors. As indispensable immune sentinels, DCs are crucial for orchestrating innate and adaptive immune responses in inflammatory, autoimmune, and malignant conditions. Highly specialized, professional antigen-presenting cells, DCs provide a multitude of necessary signals through costimulatory molecules and cytokines to T cells for their activation and differentiation. Additionally, DCs play an important role in the induction and maintenance of immunological tolerance by interacting with other immune effector cells and are considered central regulators of adaptive immune responses. Besides regulating adaptive immune responses, activated DCs are pivotal cells in immunosurveillance, spotting and destroying neoplastic cells. Tumor-infiltrating DCs (TIDCs) have been reported to have both good and poor prognostic associations in different tumor types because of their phenotypic complexities as either activated or tolerogenic DCs. In the tumor microenvironment, tolerogenic TIDCs are characterized by low expression of regulatory molecules and receptors with blunted antigen cross-presentation, leading to immunosuppression [116]. The maturation and antigen presentation functions of TIDCs can be seriously impeded by the high levels of VEGF, CCL2, CXCL1, and CXCL5, released by CAF-induced tumor cells. Binding of PD-1 with PD-L1 on the surface of DCs is upregulated by tumor cells, leading to a tolerogenic and immunosuppressive environment to undermine cytotoxic T cell activation and function [117]. The polarization of DC differentiation into regulatory DCs (rDCs) is directed by tumor-released factors that render rDCs to show protumor instead of antitumor activities by promoting regulatory T cells and myeloid-derived suppressor cells (MDSCs) in the TME. In hepatocellular carcinoma, it has been observed that CAFs can transdifferentiate TIDCs into the rDC phenotype by activating the IL-6-mediated STAT3 pathway. These rDCs are functionally disabled cells that cannot secrete T-cell-stimulatory cytokines such as IL-12, rather secreting inhibitory cytokines such as IL-10, TGFβ, and IDO and only weakly expressing costimulatory molecules [118]. In a lung cancer study, it was found that the impaired differentiation and function of DCs was due to tryptophan degradation, which was caused by both CAF-released IDO1 and lung-cancer-derived galectin-1-induced tryptophan 2,3-dioxygenase (TDO2) [119]. Studies have shown that degradation of tryptophan is associated with toxic effects on immune cells, including prevention of T cell activation and proliferation. Furthermore, research has shown that VEGF produced by CAFs through activation inhibition of NFκB contributes to reduced antigen presentation and aberrant differentiation of DCs [120]. For future DC-targeted therapy, drugs that selectively block the immunosuppressive pathways and restore the immunostimulatory function of DCs in the tumor environment hold great promise. Increasing understanding of the immunobiology of DCs, in particular regulatory DCs, and how cross-presentation of antigens by DCs improves the antitumor functions of cytotoxic T cells can help to formulate effective DC vaccines as a treatment modality in many cancers.

### 3.6. Interaction of CAFs and T Lymphocytes

T lymphocytes are exclusive, pivotal cells of adaptive immunity that include their major subpopulations, helper (Th CD4^+^), cytotoxic (CTL CD8^+^), and regulatory (Tregs) cells. CTLs are sentinels in immunosurveillance against cancers and principal cells of antitumor immunity. Profound tumor-directed cytotoxicity is exhibited by tumor-infiltrating lymphocytes (TILs), accomplished through a number of mechanisms, including production of large amounts of proinflammatory cytokines such as TNF-α and IFN-γ; secretion of death-inducing granules containing granzymes, perforin, cathepsin C, and granulysin; and apoptosis via Fas-Fas ligand (FasL) activation of caspases and endonucleases. For activation of TILs, several positive signals are provided by dendritic cells and Th cells to optimize the magnitude and capacity of the TIL-mediated cytotoxicity. Meanwhile, CD4^+^ helper T cells are pivotal immune cells in adaptive immunity, involved in both humoral (through activation and differentiation of B cells to antibody-secreting plasma cells) and cell-mediated immunity (by enhancing the capacity of CTLs and dendritic cells) towards elimination of tumor cells [121]. On the other hand, Tregs are FOXP3- and CD25-positive CD4^+^ T lymphocytes (CD4^+^CD25^+^FoxP3^+^) that promote immunosuppression and a tolerogenic tumor microenvironment by secreting inhibitory cytokines such as TGF-β and IL-10 and by interacting with other immune cells [122].

Subsets of CAF play an important role in regulating the activation and functions of all subsets of T cell in the TME. There are three important subsets of CD4^+^ Th cells, viz., Th1, Th2, and Th17, distinguished from naïve CD4^+^ T cells by different cytokine profiles and effector functions [123]. Based on production of specific cytokines, cellular and humoral immune responses are mediated by Th1 and Th2 cells, respectively, while T17 cells are associated with autoimmune diseases through the proinflammatory immune pathway [124]. Although precise mechanisms are yet to be explored, several reports have demonstrated that CAFs have a significant impact on the polarization of Th cells into different subsets in the tumor milieu. In an in vitro study on pancreatic cancer, polarization of naïve CD4^+^ T cells into the Th2 subtype was promoted by a suppressive CAF phenotype upon stimulation by the thymic stromal lymphopoietin (TSLP)-dependent pathway [125]. Interestingly, the opposite observation was made in prostate cancer, where CAF-induced lactate promoted the polarization of naïve CD4^+^ T cells into the Th1 phenotype via the activation of the micro RNA21–Toll-like receptor 8 axis [126]. Yet another study with hepatocellular CAFs demonstrated that there was low expression of costimulatory molecules on Th cells, which disabled functions of CTLs and promoted expansion of Tregs indirectly via IL-6 induced regulatory DCs [118]. The overwhelming consensus is that T cells are modulated by CAFs and transformed into immunoinhibitory subpopulations in tumors, resulting in the development of a suppressive TME.

As the most important adaptive immune cell for the antitumor immunity, CD8^+^ TILs mediate apoptosis-induced cytotoxicity to primarily cause death of tumor cells [127]. Understanding the crosstalk between CAFs and TILs in the TME is an area of research importance to reveal how the infiltration, growth, and antitumor effects of TILs are severely inhibited by CAFs [128]. Hypoxic TME is considered to be an important contributor towards CAF-mediated modification of the ECM that causes physical obstructions and restricts T cell migration and infiltration [129]. CAFs promote angiogenesis in the TME by releasing many angiogenic factors, including VEGF, in response to hypoxia, which reduces expression of endothelial cell adhesion molecules, such as intercellular adhesion molecule-1 and -2 (ICAM-1/2) and vascular cell adhesion molecule-1 (VCAM-1), seriously hampering the progression of cytotoxic T cells from peripheral blood into the tumor sites [130]. The decreased cytotoxic activity of the TILs is further accentuated by a variety of chemical factors released by FAP^+^ CAFs, including IL-6, TGFβ, and the CXCL12 signaling pathway [131]. It has been demonstrated that an TGFβ-induced ECM protein released by CAFs called TGFβ1 βig-h3 can affect TILs directly and suppress their activation, proliferation, and cytotoxic functions [132]. Further, CAFs can promote T cell anergy by expressing arginase II and galectin, which render TILs nonfunctional [133]. Indirect inhibition of antitumor activities of TILs is mediated by CAFs through different immune modulatory actions, including disruption of differentiation of DCs or NK cells, expression of immune inhibitory checkpoints, and promotion of immunoinhibitory cellular subsets such as M2 TAMs, myeloid-derived suppressor cells (MDSCs), and Treg cells [15]. Further, it is interesting to note that CAFs may assume a role like that of normal DCs in antigen presentation, processing and upregulating immune inhibition checkpoint molecules and thereby encouraging an imbalance between cytotoxic and helper T cells in the TME with an eventual immunosuppressive impact [98].

Reciprocal interaction between Treg cells and CAFs is well known, and these cells can influence each other’s differentiation in the TME. Immunosuppressive and tolerogenic tumor environments are much facilitated by the crosstalk between these two major cells. In fact, Treg cells with high Foxp3 expression and CAFs are close functionally, and both of them play important roles in controlling antitumor immunity [134]. According to clinical data, poor prognosis is strongly correlated with the simultaneous presence of both CAFs and Treg cells with high Foxp3 in the tumor stroma, indicating the possibility of crosstalk between them [135]. Infiltration and high accumulation of Treg cells in tumor sites are stimulated by CD70^+^ CAFs, as seen in colorectal cancer [136]. The CAF-released chemokine CCL5 and growth factor VEGF-A have been found necessary for Treg cell infiltration and maintenance in breast cancer [15,137]. The infiltration of Treg cells is facilitated by CAFs with downregulated CD68 induced by CCL17 and CCL22 secreted by tumor cells [138]. Additionally, differentiation of CD4^+^ naïve T cells into Treg cells is promoted by CAF-derived TGFβ through upregulation of Foxp3 gene expression in T lymphocytes, resulting in immune suppression [139]. Further, in breast cancer, CAF-S1 (FAP^+^PDGFRβ^+^) was found to be associated with the migration and transformation of CD4^+^CD25^+^ T cells into Treg cells, which are Foxp3^+^ [140]. However, an enigmatic relationship between CAFs and Treg cells was observed in pancreatic ductal adenocarcinoma (PDAC), where increased proliferation of CD4^+^Foxp3^+^ Tregs was correlated with exhaustion of myofibroblasts, resulting in inhibition of immune surveillance [141]. Increasing understanding of pivotal role of CAFs in cancer biology, including their immunoregulatory influence on the functions of T cells, could lead to progress in cancer treatment by combining immune checkpoint and CAF-targeted therapies.

### 3.7. Interaction of CAFs and Myeloid-Derived Suppressor Cells (MDSCs)

Myeloid-derived suppressor cells (MDSCs) are heterogeneous populations of immature innate immune cells characterized by their negative immune regulatory role. MDSCs are associated with cancer, chronic inflammation, autoimmunity, and stress. Under physiological condition, mature granulocytes, macrophages, or dendritic cells are generated through a quick differentiation of immature myeloid cells (IMCs). However, in pathological conditions, differentiation of IMCs into mature phenotypes is prevented, resulting in accumulation of MDSCs. Pathologically activated neutrophils and monocytes that develop into MDSC phenotypes are activated through specific signaling pathways and acquire different genomic, proteomic, and metabolic features. Cells of MDSC linage are characterized by high expression of arginase (ARG1), TGF-β, PD-L1/2, IL-10, PGE2, and IDO. Multiple mechanisms are utilized by the MDSCs for their immunosuppressive actions to downregulate the antitumor activities of immune cells, including direct inhibition of T cell activation and expansion as a major event. Morphologically, MDSCs comprise two major subpopulations, polymorphonuclear or granulocytic MDSCs (PMN-MDSCs) and monocytic MDSCs (M-MDSCs), which resemble neutrophils and monocytes, respectively, with regards to their morphology and functional phenotype. Subpopulations of MDSCs differ significantly between mice and humans in terms of surface expressing molecules. In humans, M-MDSCs are characterized by CD11b^+^CD14^+^CD33^+^HLA-DR^low/neg^, while PMN-MDSCs have a CD11b^+^CD15^+^CD14^−^CD33^+/low^HLA-DR^low/neg^ phenotype. Various cytokines produced by both tumor and stromal cells, such as GM-CSF, M-CSF, VEGF, IFN-γ, IL-4, IL-6, IL-13, and TGF-β, are involved in MDSC activation through STAT3 pathways. Each subpopulation of MDSCs has a unique ability to regulate immune responses. For example, PMN-MDSCs preferentially cause immune suppression through upregulation of reactive oxygen species, peroxynitrite, arginase 1, and PGE_2_, while M-MDSC-mediated immunosuppressive effects are caused by the release of inhibitory cytokines such as IL-10 and TGFβ and high expression of PDL1. Between these two subpopulations, M-MDSCs show higher immunosuppressive potential than PMN-MDSCs. Thus, MDSCs promote an immunosuppressive TME to facilitate angiogenesis, invasion, and metastasis for cancer through various secreted molecules and signaling pathways.

A recent report showed that circulating fibrocytes (an inactive mesenchymal cell), a novel MDSC subset, share CAFs for their phenotypic and functional characteristics, raising the possibility that MDSCs and CAFs may be related in origin [22]. CAFs are intimately related to MDSCs and induce their expansion through several CAF-derived factors such as IL-10, TGF-β, VEGF, PGE2, and PDL1, resulting in functional inhibition of effector T cells for their antitumor activities. There is evidence that CAFs recruit both types of MDSCs to tumor sites in lung squamous cell carcinoma (LSCC) by releasing CCL2, which is a crucial activator for the STAT3 signaling pathway [142]. Similar effects were found in hepatic carcinoma and triple negative breast cancers, where CAF-induced IL-6 and CXXL12, respectively, accelerated M-MDSC accumulation in the TME, which resulted in reduced production of IFNγ with serious impediment to the growth of CTLs [142]. Further, it was revealed in a recent study of esophageal squamous cell carcinoma that generation of M-MDSCs can be synergistically accentuated by CAFs via the IL-6/exosomal microRNA-21 (miR-21) STAT3 signaling pathway [143]. Although better understanding of MDSC biology is needed to open new windows of therapeutic opportunities, given that the STAT3 signaling pathway is a central regulator in tumorigenesis and the generation of M-MDSCs, there is huge potential in new cancer chemotherapies targeting the STAT3 pathway.

## 4. Interaction of CAFs and Immune Checkpoint Molecules

Immune checkpoint molecules (ICPs) are the gatekeeper of immune responses that work through ligand–receptor pairs and include both inhibitory and stimulatory checkpoint molecules. Outcomes of cancers are chiefly attributed to the antitumor activities of immune-competent cells, especially T lymphocytes. Expression of inhibitory immune checkpoint (iICP) receptors and their ligands on the surface of cellular components in the TME results in inhibition of antitumor immune responses by the immune effector cells, leading to the survival and progression of the tumor. T cell activation is a complex event participated in and regulated by both stimulatory and inhibitory immune checkpoint receptors present on the T cells. Expression of multiple inhibitory receptors on T cells makes them into exhausted and dysfunctional phenotypes with progressive deterioration of antitumor function, as seen in most advanced cancers. Examples of important iICPs expressed on the T cell surface include PD-1, PD-L1, PD-L2, CTLA-4, TIM-3 (T-cell immunoglobulin and mucin-domain 3), LAG-3 (lymphocyte-activation gene-3), TIGIT (T cell immunoglobulin and ITIM domain), and BTLA (B and T lymphocyte attenuator) [144].

According to available data, activated CAF subsets can express iICP receptor molecules to modulate immune cell function in TME directly or indirectly through upregulation of iICPs in the TME by CAF-secreted soluble mediators such as TGF-β or CXCL10, also known as interferon gamma-inducible protein 10 (IP-10). In addition, various iICP ligands found to be expressed by CAFs significantly induce T cell deactivation and exhaustion, as seen in lung cancer, melanoma, and colon tumors [145]. High expression of many of these iICPs on the surface of both immune and tumor cells in the TME can be promoted by CAFs to add further to the immunosuppression loop especially by modulating the functional phenotype of T cells. It is worth mentioning that different CAF-derived factors such as CXCL2 and CXCL5 can also contribute to the expression of iICP receptors such as PD-L1 on tumor cells in a tumor-specific fashion, implying the involvement of a number of intracellular signaling mechanisms [37,146]. Thus, it is evident from research that both direct and indirect modulation of iICPs by CAFs induce and expand immunosuppression and facilitate the growth and progression of the tumor [144]. Analyzing specific expression patterns of CAF-induced immune checkpoint molecules can help in designing strategies for tumor diagnosis and treatment. Considering the exhaustion and functional inactivation of tumor-infiltrating T cells induced by overexpression of inhibitory immune checkpoint molecules, iICP blockers as immunotherapy could be a breakthrough to overcome these inhibitory signals and restore the antitumor cytotoxic effects of T cells [147].

## 5. Role of CAFs in Remodeling the Extracellular Matrix (ECM)

Stephen Paget, an English surgeon, hypothesized the ‘seed and soil’ concept of cancer in 1889, with cancer cells as seed and stroma as soil. Until recently, there was a huge knowledge gap about the detailed architecture of the stroma, and particularly about the extracellular matrix and its role in tumor biology. The tumor microenvironment has now been explored in greater detail, encompassing the extracellular matrix and stromal cells that comprise mainly fibroblasts, vascular cells, immune and inflammatory cells, and huge amounts of noncellular components or soluble factors. Together, all the components play an essential role in cancer cell biology and predict outcomes. The ECM is composed of numerous macromolecules, including fibrin, collagen, glycoproteins, and proteoglycans, that form a complex scaffold for the anchorage and growth of environmental cells. The growth and development of a tumor alter the ECM around it both quantitively and qualitatively, which in turn contributes to tumor progression. CAFs remain the overwhelmingly predominant cell population in the ECM in most solid tumors and play a crucial role in remodeling the ECM to augment the process of tumorigenesis. Under the sustained stimulatory influence of cancer cells, activated CAFs are involved in remodeling the ECM to transform it into an ideal breeding ground for tumor progression, including metastasis of cancer cells [148].

CAFs are the most dominant stromal cell type within the TME. They act for signaling and remodeling of the ECM to create a tumor niche with much desmoplasia. They are continuously affected by tumor-secreted factors to enhance their capacity to synthesize and remodel ECM components towards tumor progression. By synthesizing numerous matrix proteins and increasing the deposition of new matrix components, CAFs significantly contribute to the biomechanical modification of the tumor matrix. The secretion of fibronectin and type I collagen and expression of matrix metalloproteinases (MMPs) including MMP-1 and MMP-3 are good examples of the highly modulating functions of CAFs in ECM remodeling. Through all these modifications, CAFs alter the structural organization and stiffness of the ECM, which not only facilitates the crosstalk among cells in the TME but produces immunosuppression that helps in tumor progression [149]. The stiffening of the ECM can be further increased by the CAF-upregulated expression and interaction of cytoskeletal regulators such as anillin (ANLN)-diaphanous-related formin-3 (DIAPH3). Additionally, ECM remodeling is regulated by the TGFβ1 released by CAFs, and the modified ECM reciprocates and favors the protumorigenic activities of CAFs [150]. In fact, in regard to their cancer-promoting characteristics, CAFs and ECM are intertwined and maintain a positive feedback loop [151].

There is growing evidence that CAF-induced ECM modification and remodeling contributes to cancer cell invasion, angiogenesis, and metastasis and the induction of an immunosuppressive TME that not only allows tumor progression but causes chemoresistance. Infiltration of immune cells, particularly T lymphocytes, into the TME is physically blocked by CAF-remodeled ECM protein networks, leading to decreased antitumor activities of immune cells [152]. The distribution of T cells in the TME is heavily predicted by the ECM collagen density, and in cases of lung and pancreatic cancers, it has been seen that there was higher collagen deposition around tumor cell islets than other parts, which physically protected cancer cells from contact with T cells [153]. Additionally, CAF-directed matrix deregulation introduces focal adhesion kinases (FAKs), nonreceptor tyrosine kinases that are extensively implicated in cancer cell growth, adhesion, migration, and ECM remodeling through their aberrant signaling. In conjunction with fibrotic regulators, FAKs play a pivotal role in the remodeling of the ECM, resulting in inadequate infiltration of CD8^+^ cytotoxic T cells [154]. The highly dynamic nature of the remodeled ECM not only favors the growth of the primary tumor but contributes efficiently to the formation of the premetastatic niche (supportive metastatic microenvironment in distant organs created by the primary tumor), which facilitates tumor metastasis. Recent studies have identified a number of ECM proteins in the premetastatic niche that play crucial roles as mediators of metastasis. In metastatic lung cancer, both primary-tumor-released factors, such as MMPs and exosomes, and CAF-secreted proinflammatory cytokines, such as TGF β1, IL-6, and IL-8, were found to enhance the formation of a premetastatic niche that helped lung metastasis of liver cancer [155,156]. The dense, fibrous stroma of the ECM surrounding tumor islets hinders cancer chemotherapy, and recent research has shown that cancer chemotherapy combined with pharmacologic FAK inhibitors can increase responsiveness to chemotherapy and immunotherapy by lowering the stromal density in various solid cancers [157]. Further, because of the fact of central role of the remodeled ECM in tumor metastasis, it has been observed that therapies that can ameliorate ECM fibrosis and integrin adhesion signaling could be an attractive adjunct to potentially cure metastatic disease [158].

Besides tumor-infiltrating T cells, CAF-modified ECM regulates other immunoinhibitory cell subpopulations in the TME, such as TAM, TAN, NK cells, TIDCs, and MDSCs [26]. For instance, monocyte migration, proliferation, and differentiation to M2 polarization were all induced by a collagen-rich matrix promoted by CAFs [12]. Reciprocally, M2 TAM contributes to increase matrix rigidity and ECM remodeling by deposition and geometrical organization of collagen. Further, recruitment and immune-inhibitory actions of Tregs, MDSCs, TAMs, and TILs have been facilitated by the CAF-induced stiffness of the fibrous stroma in the ECM through activation of aberrant FAK signaling within the cells. In conclusion, the exact mechanisms of tumor growth and metastasis are still unclear, but there is convincing evidence that remodeling of the ECM not only helps in tumorigenesis and induction of an immunosuppressive TME but favors the migration of cancer cells to develop metastasis through the formation of certain tracks or channels within the ECM [159].

## 6. CAF Signals to Tumor-Infiltrating Lymphocytes Towards Tumor Resistance

As already stated, CAFs play a significant role not only in tumor progression but in immune evasion by tumor cells through various mechanisms, including the secretion of soluble factors, remodeling of the ECM, and direct cell–cell interactions in the tumor microenvironment. All these signals are crucial in the susceptibility or resistance of tumor cells to killing by TILs. The following CAF-mediated mechanisms are thought to be involved in regulating the cytotoxic activities of TILs, leading to tumor resistance.

### 6.1. Secretion of Immunosuppressive Cytokines

Transforming growth factor beta (TGF-β): CAF-derived TGF-β inhibits the activation of TILs and impairs their ability to induce apoptosis through cytotoxic molecules such as perforin, granzyme B, and IFN-γ in tumor cells [160,161].

Interleukin-10 (IL-10): Production of proinflammatory cytokines such as IL-2 and IFN-γ by TILs is seriously impeded by IL-10, resulting in suppression of the cytotoxic activity of TILs [38].

### 6.2. Modulation of Immune Checkpoints

Programmed death-ligand 1 (PD-L1): CAF-mediated cytokines such as IL-6 and IL-8 can upregulate the expression of PD-L1 on tumor cells, and interaction of PD-L1 and PD-1 on TILs leads to TIL exhaustion, characterized by reduced cytotoxic activity and diminished production of effector molecules [162,163].

Other immune checkpoints: Tumor cells can also exploit other checkpoint molecules such as CTLA-4, TIM-3, and LAG-3 to inhibit TIL activity and promote resistance [162].

### 6.3. Metabolic Adaptation and Competition

Tumor metabolism: Tumor cells can alter their metabolic pathways to create a microenvironment low in glucose and high in lactate, which can impair TIL function. Tumor cells with high glycolytic rates can outcompete TILs for nutrients, reducing TIL effectiveness [145].

Immune checkpoint metabolism: CAFs can produce enzymes such as arginase and IDO that deplete essential nutrients such as L-arginine and tryptophan from the tumor microenvironment. This metabolic competition impairs TIL proliferation and function, including the production of cytotoxic granules [145].

### 6.4. Altered Trafficking by Secretion of Chemokines

CXCL12 (SDF-1): CXCL12 can act as a chemoattractant to the tumor site for immune suppressor cells such as myeloid-derived suppressor cells (MDSCs) and Tregs, which in turn suppress TIL function. It can also alter TIL trafficking, leading to its sequestration away from tumor cells [164,165].

### 6.5. Alterations in Apoptotic Pathways

Antiapoptotic proteins: Tumor cells can overexpress antiapoptotic proteins such as Bcl-2, Bcl-xL, and Mcl-1, which inhibit apoptotic pathways and confer resistance to TIL-induced apoptosis [46].

Death receptor pathways: By downregulating death receptors (e.g., Fas) or upregulating decoy receptors, tumor cells can render them less susceptible to TIL-mediated cytotoxic signals [46].

### 6.6. Antigen Presentation

MHC class I downregulation: Tumor cells may reduce the expression of MHC class I molecules via IL-2 and IL-6 produced by CAFs, thereby evading recognition and killing by TILs [38].

Antigen processing machinery (APM): Defects in components of the APM, such as TAP and proteasomes, can result in inefficient peptide loading onto MHC class I molecules, reducing the visibility of tumor antigens to TILs [38].

### 6.7. Remodelling of the Extracellular Matrix (ECM)

Production of dense ECM: CAFs can physically hinder the movement of TILs by producing a dense ECM, reducing their ability to contact and kill tumor cells effectively [15].

ECM-associated proteins: ECM proteins such as fibronectin and collagen can bind directly to receptors on TILs (e.g., integrins), reducing their signaling and cytotoxic potential [15].

### 6.8. Expression of Inhibitory Ligands

Galectins and other ligands: CAFs can express molecules such as galectin-1 that bind to glycosylated receptors on TILs, inducing apoptosis or functional impairment of the TILs [166].

### 6.9. Direct Cell–Cell Interaction

Adhesion molecules: CAFs can interact directly with tumor cells via adhesion molecules such as N-cadherin, ICAM-1, VCAM-1, etc. and send inhibitory signals to TILs, dampening their cytotoxic responses [38].

## 7. Immunotherapeutic Strategies for Tumors Based on Modulation of CAFs

The susceptibility or resistance of tumor cells to TIL-mediated killing is a complex interplay of immune evasion strategies, metabolic adaptations, and modulation of the tumor microenvironment where CAFs remain the most important players. Understanding these mechanisms allows for the development of CAF-targeted therapies to overcome resistance and enhance the effectiveness of TILs in cancer treatment [167].

Table 1 summarizes strategies leveraging CAFs for tumor suppression and tumor cell death with mechanisms, approaches, tumor types, and clinical status.

## 8. Conclusions

The biology of CAFs is complex, and the heterogeneity in origins and functional phenotypes of unique CAF subpopulations in various tumor types has yet to be explored. However, as an integral component of the TME, CAFs remain highly versatile, plastic, and resilient and play indispensable roles in tumorigenesis and metastasis. CAFs have been found in both primary and metastatic tumor locations as the most abundant stromal cells that interact with all available cell types in the TME through various secreted molecules and signaling pathways for the progression of cancer. Tumor-integrated processes such as angiogenesis, immune response, and metabolism are significantly altered by epigenetically controlled CAF-secreted exosomes and metabolites. Evidence from numerous studies supports the fact that in a context-dependent manner, CAF subtypes can impart both tumor-promoting and tumor-restraining actions. Although less discussed, CAFs have been found to exhibit antitumor activities through secretion of proinflammatory cytokines, inhibitory signaling, and modulation of ECM components that collectively show inhibitory effects to tumorigenesis and metastasis [173]. Certain myCAF (αSMA^+^) subpopulations have demonstrated tumor-restraining functions in some cancers when stimulated by the Sonic hedgehog (SHH)–smoothened (Smo) signaling pathway, a critical cell–cell communication system for adult tissue homeostasis. In preclinical models of pancreatic cancer, it was observed that an increased ratio of myCAF to iCAF induced by leukemia inhibitory factor (LIF) inhibited cancer cell proliferation through deposition of ECM proteins that in turn resulted in regression of tumor growth. Additionally, deposition of type I collagen by αSMA^+^ myofibroblasts has been linked to their tumor-restraining functions.

Because of their multifaceted roles in tumorigenesis, CAFs constitute a potential therapeutic target in cancer immunotherapy strategies. CAF-targeted immunotherapies include four general strategies/targets in CAFs: (i) fibroblast activating protein (FAP), (ii) activators and signaling pathways, (iii) reprogramming to dedifferentiate activated CAFs, and (iv) stromal depletion [174]. Until recently, clinical trials with CAF-targeted therapies have been unsuccessful, possibly because of their tumor-restraining functions. Because of the functional heterogeneity of subtypes of CAFs, specific targets for the tumor-promoting subtype are difficult to find because (a) CAFs lack definitive biomarkers and signaling pathways; (b) nonbinary features exist between CAF subpopulations; and (c) CAFs are likely to be both tumor-promoting and tumor-retarding cells. Thus, the clinical relevance of CAFs in terms of therapeutic strategies and prognostic value demands reevaluation and redefinition according to the distribution of distinct subpopulations of CAF and their roles in different cancers. It is generally agreed that CAF-targeted therapies together with checkpoint blockade immunotherapies hold more promise for tumors rich in CAFs [173].

In this narrative review, a concise overview is provided of the biology of CAFs and their roles in modulating immune effector cells towards an immunosuppressive TME to augment tumor growth and metastasis. Precise biological understanding of crosstalk between CAFs and immunocompetent cells in the TME holds great promise for CAF-targeted immunotherapies of solid tumors, especially when combined with checkpoint blockers. However, several concerns need to be resolved before the translation of such therapies from bench to bedside. Development of novel antibodies targeting specific subtypes of CAF based on specific markers could pave the way to differentiating functional protumor CAFs from antitumor subtypes. Only then can antibody drug conjugates as a CAF-targeted modality bring treatment success in cancers. Until recently, most of the research on CAFs has been confined to animal models or limited to preclinical trials, and there is still a long way to go to translate these facts into clinical applications. Identifying CAF-specific gene expression signatures and specific protein markers based on tumor types through single-cell transcriptome analyses (single-cell RNA sequencing data), lineage tracing, immunophenotyping, and spatial architectural analysis of TME could aid in comprehending the traits and plasticity of CAFs and may offer further fresh perspectives on the biological understanding and usefulness of CAFs. Future approaches may include identification of changes in both stromal cells and immune cells that occur during tumor progression through new and emerging technologies such as single-cell transcriptomics (scRNA-seq), spatial transcriptomics (to study intact tissue), or protein profiling in tissue to evaluate spatial changes in the TME.

## Figures and Tables

**Table 1:** Strategies leveraging CAFs for tumor suppression and tumor cell death.

Strategy	Mechanism	Therapeutic Agents/Approach	Tumor Type(s)	Clinical Status	References
**CAF reprogramming to antitumor phenotype**	Converts tumor-promoting CAFs to tumor-suppressive phenotypes	- All-trans retinoic acid (ATRA) - Vitamin D analogs (e.g., calcipotriol)	Pancreatic, breast	Preclinical–Phase I	[168]
**CAF-induced immune activation**	Enhances CAF antigen presentation to stimulate T cell activity	- CD40 agonists - IL-12-expressing vectors in CAFs	Pancreatic, melanoma	Preclinical	[169]
**CAF-mediated delivery of cytotoxic agents**	Engineers CAFs to produce or deliver drugs locally	- CAF-loaded nanoparticles - CAF-targeted viruses (e.g., FAP promoter-controlled adenovirus)	Solid tumors	Preclinical	[170]
**Promotion of tumor vessel normalization**	Targets CAF signaling to normalize vasculature	- TGF-β inhibitors (e.g., galunisertib) - Angiotensin receptor blockers (ARBs)	Breast, colorectal, pancreatic	Phase I–II	[171]
**CAF-targeted senescence induction**	Induces senescence in tumor-promoting CAFs	- CDK4/6 inhibitors (e.g., palbociclib)	Breast, pancreatic	Preclinical	[172]
**Restoring ECM homeostasis via CAFs**	CAF modulation reduces excessive ECM stiffness and desmoplasia	- PEGPH20 (hyaluronidase) - Lysyl oxidase inhibitors	Pancreatic, liver	Phase II–III	[37]
**CAF–immune cell cotargeting**	Concurrently modulates CAFs and immune cells for synergistic tumor killing	- CXCR4 inhibitors + checkpoint blockade - CAF vaccine + T cell therapy	Pancreatic, lung, melanoma	Early clinical trials	[170]

## Data Availability

All data are in the manuscript.

## Abbreviations

CAFs	cancer-associated fibroblasts
TME	tumor microenvironment
ECM	extracellular matrix
Treg	T regulatory cells
MDSCs	myeloid-derived suppressor cells
FAP	fibroblast activation protein
SDF-1	stromal-derived factor-1
VEGF	vascular endothelial growth factor
PGE2	prostaglandin E2
C-C	chemokine ligand 2
IDO	indoleamine 2,3-dioxygenase
TAMs	tumor-associated macrophages
TANs	tumor-associated neutrophils
DCs	dendritic cells
rDCs	regulatory dendritic cells
TILs	tumor-infiltrating lymphocytes
TIDCs	tumor-infiltrating DCs
MSCs	mesenchymal stem cells
BMSCs	bone marrow mesenchymal stem cells
EMT	epithelial–mesenchymal transition
TGFβ1	transforming growth factor β1
ADSCs	adipose tissue-derived mesenchymal stem cells
FSP-1	fibroblast specific protein 1
EndMT	endothelial–mesenchymal transition
α SMA	α smooth muscle actin
MMT	mesothelial-mesenchymal transition
PSCs	pancreatic stellate cells
PDGF	platelet-derived growth factor
FGF-2	fibroblast growth factor 2
HGF	hepatocyte growth factor
ROS	reactive oxygen species
CCL	C-C chemokine ligands
JAK/STAT3	Janus kinase/TNF inducer and activator of transcription 3
DAMPs	damage-associated molecular patterns
VIM	vimentin
CAV-1	*caveolin-1*
myCAFs	myofibroblast CAFs
iCAFs	inflammatory CAFs
apCAF	antigen-presenting CAFs
PDA	pancreatic ductal adenocarcinoma
scRNA-seq	single-cell RNA sequencing
MCP-1	monocyte chemotactic protein-1
Chi3L1	chitinase 3-like protein 1
PD-1	programmed cell death protein 1
NLR	neutrophil-to-lymphocyte ratio
ICAM1	intercellular adhesion molecule 1
VCAM-1	vascular cell adhesion molecule-1
MPO	myeloperoxidase
HCC	hepatocellular carcinoma
SCF	stem cell factor
TSLP	thymic stromal lymphopoietin
LSCC	lung squamous cell carcinoma
miR-21	microRNA-21
iICPs	immune checkpoints
MMPs	matrix metalloproteinases
FAKs	focal adhesion kinases
SHH	Sonic hedgehog
Smo	smoothened
